# Effects of different external cooling placements prior to and during exercise on athletic performance in the heat: A systematic review and meta-analysis

**DOI:** 10.3389/fphys.2022.1091228

**Published:** 2023-01-10

**Authors:** Dongting Jiang, Qiuyu Yu, Meng Liu, Jinjin Dai

**Affiliations:** ^1^ Sports Coaching College, Beijing Sports University, Beijing, China; ^2^ Big Ball Sports Center, Hebei Provincial Sports Bureau, Shijiazhuang, China

**Keywords:** external cooling, cooling placements, athletic performance, heat, hot environment

## Abstract

**Background:** Nowadays, many high-profile international sport events are often held in warm or hot environments, hence, it is inevitable for these elite athletes to be prepared for the challenges from the heat. Owing to internal cooling may cause gastrointestinal discomfort to athletes, external cooling technique seems to be a more applicable method to deal with thermal stress. Central cooling mainly refers to head, face, neck and torso cooling, can help to reduce skin temperature and relieve thermal perception. Peripheral cooling mainly refers to four limbs cooling, can help to mitigate metabolic heat from muscular contrac to effectively prevent the accumulation of body heat. Hence, we performed a meta-analysis to assess the effectiveness of different external cooling placements on athletic performance in the heat

**Methods:** A literatures search was conducted using Web of Science, MEDLINE and SPORTDiscus until September 2022. The quality and risk of bias in the studies were independently assessed by two researchers.

**Results:** 1,430 articles were initially identified (Web of Science = 775; MEDLINE = 358; SPORTDiscus = 271; Additional records identified through other sources = 26), 60 articles (82 experiments) met the inclusion criteria and were included in the final analysis, with overall article quality being deemed moderate. Central cooling (SMD = 0.43, 95% CI 0.27 to 0.58, *p* < 0.001) was most effective in improving athletic performance in the heat, followed by central and peripheral cooling (SMD = 0.38, 95% CI 0.23 to 0.54, *p* < 0.001), AND peripheral cooling (SMD = 0.32, 95% CI 0.07 to 0.57, *p* = 0.013). For the cooling-promotion effects on different sports types, the ranking order in central cooling was ETE (exercise to exhaustion), TT (time-trial), EWT (exercise within the fixed time or sets), IS (intermittent sprint); the ranking order in peripheral cooling was EWT, TT, ETE and IS; the ranking order in central and peripheral cooling was ETE, IS, EWT and TT.

**Conclusion:** Central cooling appears to be an more effective intervention to enhance performance in hot conditions through improvements of skin temperature and thermal sensation, compared to other external cooling strategies. The enhancement effects of peripheral cooling require sufficient re-warming, otherwise it will be trivial. Although, central and peripheral cooling seems to retain advantages from central cooling, as many factors may influence the effects of peripheral cooling to offset the positive effects from central cooling, the question about whether central and peripheral cooling method is better than an isolated cooling technique is still uncertain and needs more researchs to explore it.

## Introduction

During the period of exercise, about more than 75% energy that is generated by skeletal muscle substrate oxidation is liberated as heat, which inevitably lead to an increase in body temperature (Wendt et al., 2007). However, The heat dissipation capacity of the human body would be impaired resulting in a further increase in body temperature, when coupled with hot environmental conditions (Berggren and Hohwu Christensen, 1950; Saltin and Hermansen, 1966). Nowadays, many high-profile international sport events are often held in warm or hot environments, such as 2022 Federation International Football Association (FIFA) World Championship in Qatar, 2023 International Association of Athletics Federations (IAAF) World Championships in Budapest, and 2024 Olympic Games in Paris. These hot environmental conditions would pose a major challenge to the physiological status and sport performance of athletes during the competition, and even lead to fatal incidents (Casa et al., 2005; Vanos et al., 2018). Hence, it is inevitable for these elite athletes to be prepared for the challenges from the heat.

Pre-cooling is considered as a practical on-field strategy for athletes to relieve the adverse effects from heat-stress-induced fatigue and enhance athletic performance, because it is easy to be implemented during the warm-up period (Hohenauer et al., 2018). According to the application of the cooling technique used, cooling strategies are categorized into internal cooling strategy (e.g., cold water ingestion, ice slurry ingestion, etc) and external cooling strategy (e.g., ice vest, ice packs, cold water immersion, etc) (Ross et al., 2013). However, while the effect of ice slurry ingestion on reducing core temperature is significant, it may cause gastrointestinal discomfort to athletes, especially those with gastrointestinal diseases (Bongers et al., 2017). For this reason, external cooling technique instead of internal cooling technique may be a relatively safer and more applicable method to deal with thermal stress. Because in addition to cutaneous thermoreception, thermoreceptive mechanisms exist in body core structures including the brain, spinal cord and abdomen (Morrison and Nakamura, 2011), and an elevated core body temperature is the critical limiting factor for exercise performance in the heat (Maughan, 2010). Hence, central cooling (face, neck, head and torso cooling) may have a relatively more direct effect on mitigating physiological strains and thermal perception to promote athletic performance in the heat (Cotter and Taylor, 2005). On the other hand, peripheral cooling (four limbs cooling) can help to mitigate metabolic heat from muscular contraction to effectively prevent the accumulation of body heat, and improve athletic performance in the heat by the maintenance of muscle recruitment (Randall et al., 2015). Thus, the question about the most effective external cooling placement (central? Or peripheral?) remains unclear.

This study thus aims to quantitatively examine the effects of different external cooling placements on athletic performance in the heat (≥ 28°C) in athlete by completing a systematic review and meta-analysis of the peer-reviewed publications. For practical-application considerations, we conducted subgroup analysis of exercise protocols to explore the influences of each external cooling intervention on various exercise types, which may provide valuable lessons for athletes to select targeted external cooling strategies when performing diverse exercise types (e.g., sustained endurance exercise, intermittent sprint exercise, etc) under hot thermal conditions. Finally, we also proposed some potential research issues about practical external cooling strategies in the future.

## Materials and methods

This systematic review and meta-analysis was conducted using Preferred Reporting Items for Systematic Reviews and Meta-Analysis guidelines (PRISMA) (Page et al., 2021) and registered with PROSPERO (Registration ID CRD42022368476), an international prospective registry for systematic reviews.

### Data sources and search strategy

Two authors (DJ and QY) independently searched Web of Science, MEDLINE and SPORTDiscus data bases from inception to September 25th 2022, using a comprehensive search strategy (Supplementary Table S1). Manual searches of the reference lists in the related publications were also performed.

A researcher (QY) imported all records into EndnoteX9 software and deleted any duplicates. Then, two researchers (DJ and JD) checked the title and abstract separately to exclude any unrelated articles, with the remaining full texts screened against the inclusion criteria. Discrepancies over article inclusion were settled through discussion with a third researcher (ML) until consensus was achieved. Only peer-reviewed parallel and crossover randomized controlled trials (RCTs) written in English were classifed as relevant. Based on the information within the full text, the inclusion and exclusion criteria were applied to select the trials eligible for inclusion in the review and meta-analysis. Conference papers and case studies were excluded, as were reviews, but their references were manually screened to ensure all appropriate citations were also considered for inclusion.

### Eligibility criteria

A PICOS (Participants, Intervention, Comparators, Outcomes, and Study design) approach was used to rate studies for eligibility (Liberati et al., 2009). Studies were eligible if they met the following criteria: 1) the design of study was parallel or crossover RCT; 2) subjects included were healthy and non-injured humans without heat acclimation; 3) male and female study participants were included; 4) hot ambient conditions (≥ 28°C); 5) cooling was conducted prior to and during exercise, which may have included recovery cooling where subjects cooled after one bout of exercise (e.g., simulation of halftime) and before a second bout; 6) cooling interventions were limited to external cooling; 7) specific cooling placements were mentioned; 8) control interventions were passive non-cooling strategies; 9) measurement of athletic performance; 10) provided full data (mean and SD) that allowed effect sizes to be calculated; 11) the manuscripts were written in English and were published in a peer-reviewed journal. Articles were excluded if: 1) studies were incomplete (e.g., abstracts); 2) outcome measures were merely based on physiological parameters; 3) repeated publications.

### Data extraction and variable categorisation

The two researchers (QY and JD) extracted the data from the selected literatures with a standard table. In case of disagreement, a third researcher (DJ) checked the data in the original study and agreement was sought by consensus. The following data were extracted: studies (author, year), sample size (number, sex), ambient conditions (temperature, relative humidity), cooling strategies (type, placement, method), exercise details (type, modality, protocol) and sport performance outcome measures. When one study consisted of multiple sport performance outcome measures, only one outcome measure was used and included in the analysis. Discrepancies over sport performance outcome were settled through discussion with a third researcher (ML) until consensus was achieved. When studies only reported standard errors, standard deviations were calculated by multiplying the standard error by the square root of the sample size (Altman and Bland, 2005). In addition, when studies only reported split results, the total results were were calculated with the following algorithms (Higgins et al., 2019):

$$N_{pooled}=N_{1}+N_{2}$$

$${Mean}_{pooled}=\frac{N_{1}M_{1}+N_{2}M_{2}}{N_{1}+N_{2}}$$

$${SD}_{pooled}=\sqrt{\frac{\left(N_{1}-1\right){{SD}_{1}}^{2}+\left(N_{2}-1\right){{SD}_{2}}^{2}+\frac{N_{1}N_{2}}{N_{1}+N_{2}}\left({M_{1}}^{2}+{M_{2}}^{2}-2M_{1}M_{2}\right)}{N_{1}+N_{2}-1}}$$

In the event that relevant data were not available, the corresponding authors of the manuscripts were contacted to request it (Higgins et al., 2019). Studies with missing data that could not be retrieved or provided by the author were excluded from the meta-analysis.

The reviewed articles were divided into three groups depending on the different external cooling placements, namely: central cooling, peripheral cooling and central and peripheral cooling (Giesbrecht et al., 1995; Drinkwater, 2008; Rodríguez et al., 2020). Based on the basic characteristics of different exercise protocols, subsequent analysis was conducted on the external cooling data by dividing the data set into four groups: 1) intermittent sprint (IS); 2) exercise to exhaustion (ETE); 3) exercise within fixed the fixed time or sets (EWT); 4) time-trial (TT) (Alhadad et al., 2019).

#### Quality assessment

Quality assessment was performed using the Physiotherapy Evidence Database (PEDro) Scale, which is a valid measure of the methodological quality of clinical trials (De Morton, 2009). Two researchers (DJ and QY) assessed the quality of included studies independently according to 11 items on the PEDro scale. For each item whose criterion was met, one point was awarded, whereas no points were given if the item was not fulfilled (Maher et al., 2003). The item 1 (eligibility criteria) which refers to external validity was not included in the total score calculation, and the remaining 10 items which refers to internal validity and statistical reporting were included in the total score calculation. Studies were considered high quality if PEDro scores were greater than 7, medium quality if five to six, and low quality if four and below (Cashin and McAuley, 2019). Two researchers (DJ and QY) applied the PEDro scale to evaluated the 60 studies (82 experiments) independently, and any scoring discrepancies were discussed with a third researcher (ML) until aconsensus was achieved.

### Data synthesis

To determine the effect size (ES) of the intervention, the standardized mean difference (SMD; Hedges’ g) of the outcomes was calculated, with a 95% confidence interval (CI). ES was classified as trivial (<0.2), small (0.2–.49), moderate (0.5–0.79), or large (> 0.8) (Cohen, 2013). Meta-analysis was performed in Stata 14.0 (STATA Corp., College Station, TX) using the inverse variance method. The heterogeneity was assessed by measuring the inconsistency (*I* ^2^ statistic) of intervention effects among the trials. The level of heterogeneity was interpreted according to guidelines from the Cochrane Collaboration: trivial (< 25%), low (25–50%), moderate (50–75%), and high (> 75%) (Higgins et al., 2003). The random effect model was used if there was significant heterogeneity across studies (I^2^ > 50%). Otherwise, the fixed effect model was used. The publication bias was assessed by the funnel plot and Egger’s test. Subgroup analysis was used to analyze the possible sources of heterogeneity. If a significant asymmetry was detected, we used the Trim and Fill method for sensitivity analysis of the results (Duval and Tweedie, 2000). All the statistical significance was set at *p* < 0.05.

## Results

### Search results

**FIGURE 1:**
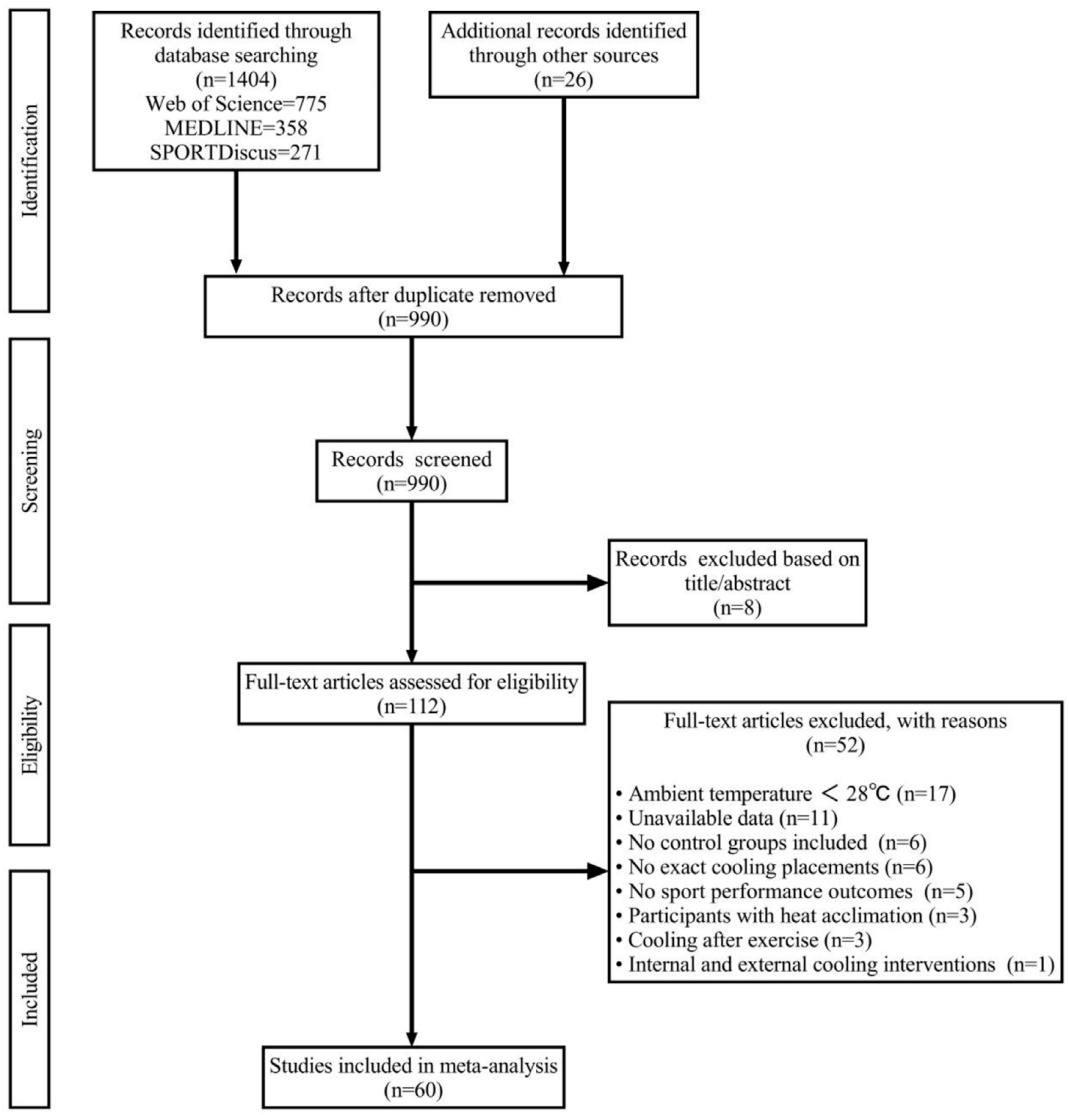
Overview of selection process of the included studies for this meta-analysis.

The initial search resulted in 1,430 records (Web of Science = 775; MEDLINE = 358; SPORTDiscus = 271; Additional records identified through other sources = 26). After excluding 440 duplicates, 990 studies were selected to be screened by title and abstract. According to the inclusion criteria, 878 papers were excluded. Subsequently, the remaining 112 full-text articles were evaluated for eligibility, and 52 of them were excluded because the ambient temperature was less than 28°C (*n* = 17), the complete raw data was unavailable (*n* = 11), not including control groups with passive non-cooling strategies (*n* = 6), not mentioning specific cooling placements (*n* = 6), not including sport performance outcomes (*n* = 5), participants were acclimatized to heat (*n* = 3), cooling interventions were conducted after exercise (*n* = 3), cooling interventions included internal and external cooling (*n* = 1). Finally, 60 articles (82 experiments) meeting the eligibility criteria were included for qualitative analysis (Figure 1).

### Quality assessment

In accordance with the PEDro, the scores of 82 in cluded experiments ranged from 5 to 7 (Supplementary Table S2), the mean score was 6.7. Hence, the overall quality of the included experiments was good. The majority of the experiments were rated as high-quality (*n* = 56), and the remaining experiments were rated as medium-quality (*n* = 26). 21 experiments did not clearly describe the specific eligibility criteria. Two experiments (Randall et al., 2015a; Randall et al., 2015b) did not get score in item eight and nine because that both only partial subjects (≤ 75%) completed all conditions to get sport performance outcome measures and not all results (≤ 75%) were included in analysis. Due to their crossover design, none of the studies could conceal allocations and blind participants from the interventions. Moreover, none of them blinded therapists or assessors (Rodríguez et al., 2020).

### Characteristics of included studies

The basic informations of the participants, ambient conditions, cooling interventions and exercise-test protocol were included in Table 1. Overall, 595 subjects were included in the qualitative analysis. The ambient temperature in the included studies varied from 29°C to 42°C and relative humidity varied from 15% to 80%. The external cooling placements of included studies mainly referred to head, neck, torso, upper limbs and lower limps. According to different external cooling placements, all external cooling interventions can be categorized into central cooling, peripheral cooling and central and peripheral cooling. Among them, 34 experiments used central cooling, 14 experiments used peripheral cooling and 34 experiments used central and peripheral cooling. Regarding cooling methods, CWI and ice vest were the most commonly used means of external cooling, overall 54 experiments’ cooling strategies involved with them. Moreover, other external cooling strategies consisted of neck-cooling device, ice towels, ice packs, cooling garment, cooling jacket, neck-cooling collar, cooling glove, water-perfused cuffs, palm-cooling device, external-pouring cold water, cooling cap, cooling shirt, fan, wet sponge, hand-cooling device, head-cooling device, heat emergency kit, hybrid cooling vest, ice bath, ice headband, ice wraps and mixed external cooling methods. Among them, 65 experiments purely utilized a single external cooling method and 17 experiments used mixed external cooling methods. Exercise modality comprised of cycling (47 experiments), running (33 experiments), rowing (1 experiment) and running combined with bench press (1 experiment). Based on the basic characteristics of different exercise protocols, they can be classified as four various exercise types (IS, ETE, EWT and TT). Specifically, 25 experiments chose IS as sport performance test, 20 experiments chose ETE as sport performance test, 20 experiments chose EWT as sport performance test and 17 experiments chose TT as sport performance test. The sport performance outcome indicators of the 82 included experiments were various. Concretely speaking, the sequence of from high to low of usage frequency of outcome indicators is: distance (19 experiments), total work done (14 experiments), duration (18 experiments), MPO (11 experiments), time (15 experiments), RPO (2 experiments), PPO (1 experiment), speed at VO2max (1 experiment), total reps (1 experiment).

**TABLE 1:** Characteristics of the included studies.

Study	Sample size	Ambient conditions	Cooling			Exercise			outcome	*p*
			type	placement	method	modality	type	protocol		
Sleivert et al. (2001)	9 males	33°C 60%RH	①	torso	ice vest	cycling	EWT	45s time-trial	MPO (W)	↓
Sleivert et al. (2001)	9 males	33°C 60%RH	③	torso thighs	ice vest & water-perfused cuffs	cycling	EWT	45s time-trial	MPO (W)	↓*
Cotter et al. (2001)	9 males	35°C60%RH	①	torso	ice vest	cycling	EWT	20min at 65%VO2peak & 15min time-trial	RPO (W·kg^-1^)	↑**
Cotter et al. (2001)	9 males	35°C 60%RH	③	torso thighs	ice vest & water-perfused cuffs	cycling	EWT	20min at 65%VO2peak & 15min time-trial	RPO (W·kg^-1^)	↑**
Hasegawa et al. (2005)	9 males	32°C 70-80%RH	①	torso	ice vest	cycling	ETE	60min at 60%VO2max & exercise to exhaustion at 80%VO2max	duration (s)	↑**
Hsu et al. (2005)	8 males	32°C 24%RH	②	palms	hand-cooling device	cycling	TT	30km time-trial	time (min)	↑**
Castle et al. (2006)	12 males	34°C 52%RH	①	torso	ice vest	cycling	IS	20×2min intermittent sprint	total work done (kJ)	↑**
Castle et al. (2006)	12 males	34°C 52%RH	③	torso four limbs	CWI	cycling	IS	20×2min intermittent sprint	total work done (kJ)	↑
Castle et al. (2006)	12 males	34°C 52%RH	②	thighs	ice packs	cycling	IS	20×2min intermittent sprint	total work done (kJ)	↑**
Hasegawa et al. (2006)	9 males	32°C	③	torso four limbs	CWI	cycling	ETE	60min at 60%VO2max & exercise to exhaustion at 80%VO2max	duration (s)	↑**
		80%RH								
Duffield and Marino (2007)	9 males	32°C	①	torso	ice vest	running	IS	2×30min intermittent sprint	distance (m)	↑
		30%RH								
Duffield and Marino (2007)	9 males	32°C	③	torso four limbs	ice vest & ice bath	running	IS	2×30min intermittent sprint	distance (m)	↑
		30%RH								
Vaile et al. (2008)	10 males	34°C	③	torso four limbs	CWI	cycling	EWT	2×(15min at 75%PPO & 15min time-trial)	total work done (kJ)	↑
		39%RH								
Vaile et al. (2008)	10 males	34°C	③	torso four limbs	CWI	cycling	EWT	2×(15min at 75%PPO & 15min time-trial)	total work done (kJ)	↑
		39%RH								
Vaile et al. (2008)	10 males	34°C	③	torso	CWI	cycling	EWT	2×(15min at 75%PPO & 15min time-trial)	total work done (kJ)	↑
		39%RH		four limbs						
Vaile et al. (2008)	10 males	34°C	③	torso	CWI	cycling	EWT	2×(15min at 75%PPO & 15min time-trial)	total work done (kJ)	↑
		39%RH		four limbs						
Duffield et al. (2009)	7 males	32°C	③	neck torso thighs	ice vest & ice towels & ice packs	running	IS	4×5min intermittent sprint	distance (km)	↑*
		44%RH								
Bogerd et al. (2010)	8 males	29°C	①	torso	cooling shirt	cycling	ETE	exercise to exhaustion at 65%VO2peak	duration (s)	↑*
		80%RH								
Bogerd et al. (2010)	8 males	29°C	①	torso	ice vest	cycling	ETE	exercise to exhaustion at 65%VO2peak	duration (s)	↑*
		80%RH								
Duffield et al. (2010)	8 males	33°C	②	lower limbs	CWI	cycling	EWT	40min time-trial	distance (km)	↑*
		50%RH								
Peiffer al. (2010)	10 males	35°C 40%RH	③	torso four limbs	CWI	cycling	TT	2×(25min at 65%VO2max & 4km time-trial)	time (min)	↑*
Tyler et al. (2010)	9 males	30°C 53%RH	①	neck	neck-cooling device	running	EWT	75min at 60%VO2max & 15min time-trial	distance (m)	↑*
Tyler et al. (2010)	8 males	30°C 53%RH	①	neck	neck-cooling device	running	EWT	15min time-trial	distance (m)	↑
Castle et al. (2011)	8 males	33°C 52%RH	②	thighs	ice packs	cycling	IS	20×2min intermittent sprint	total work done (kJ)	↔
Clarke et al. (2011)	12 males	31°C 42%RH	①	torso	ice vest	running	ETE	90min football-specific exercise & exercise to exhaustion at 8mph and at an incline of 20%	duration (s)	↑
Duffield et al. (2011)	6 males and 2 females	35°C 55%RH	③	head neck torso arms	ice vest & ice towels	running	IS	5×5min tennis-specific intermittent sprint	distance (m)	↑
Minett et al. (2011)	10 males	33°C 33%RH	①	head neak	ice towels	running	IS	2×35min intermittent sprint	distance (m)	↑
Minett et al. (2011)	10 males	33°C 33%RH	③	head neak hands	CWI & ice towels	running	IS	2×35min intermittent sprint	distance (m)	↑*
Minett et al. (2011)	10 males	33°C 33%RH	③	head neak torso hands thighs	CWI & ice vest & ice towels & ice packs	running	IS	2×35min intermittent sprint	distance (m)	↑**
Minniti et al. (2011)	8 males	30°C 53%RH	①	neck	neck-cooling device	running	EWT	75min at 60%VO2max & 15min time-trial	distance (m)	↑**
Tyler and Sunderland (2011)	7 males	30°C 53%RH	①	neck	neck-cooling device	running	EWT	75min at 60%VO2max & 15min time-trial	distance (m)	↑**
Tyler and Sunderland (2011)	7 males	30°C 53%RH	①	neck	neck-cooling device	running	EWT	75min at 60%VO2max & 15min time-trial	distance (m)	↑**
Tyler and Sunderland (2011)	8 males	32°C 53%RH	①	neck	neck-cooling device	running	ETE	exercise to exhaustion at 70%VO2peak	duration (min)	↑**
Vaile et al. (2011)	10 males	33°C 44%RH	③	torso four limbs	CWI	cycling	EWT	2×(15min at 75%PPO & 15min time-trial)	total work done (kJ)	↑*
Grahn et al. (2012)	8 males	42°C 20-35%RH	②	palm	palm-cooling device	running & bench press	EWT	exercise at 5.63km·h^-1^ and at an individual incline & 4 sets of fixed load bench press	total reps (n)	↑**
Luomala et al. (2012)	7 males	30°C 40%RH	①	torso	ice vest	cycling	ETE	exercise to exhaustion (9min at 60%VO2max & 1min at 80%VO2max)	duration (s)	↑*
Minett et al. (2012)	8 males	33°C 34%RH	③	head neak shoulders torso hands thighs	CWI & ice vest & ice towels & ice packs	running	IS	2×35min intermittent sprint	distance (m)	↔
Minett et al. (2012)	8 males	33°C 34%RH	③	head neak shoulders torso hands thighs	CWI & ice vest & ice towels & ice packs	running	IS	2×35min intermittent sprint	distance (m)	↑**
Munoz et al. (2012)	10 males	33°C 30%RH	①	head	cold water (externl-pouring)	running	TT	90min at 30%VO2max & 5km time-trial	time (min)	↑
Skein et al. (2012)	10 males	31°C 33%RH	③	torso four limbs	CWI	running	IS	50min intermittent sprint	distance (m)	↑
Scheadler et al. (2013)	12 subjects	30°C 50%RH	②	plam	palm-cooling device	running	ETE	exercise to exhaustion at 75%VO2peak	duration (min)	↓*
Brade et al. (2014)	12 males	35°C 60%RH	①	torso	cooling jacket	cycling	IS	2×30min intermittent sprint	total work done (kJ)	↑
De Pauw et al. (2014)	9 males	30°C 50%RH	③	torso four limbs	CWI	cycling	TT	60min at 55%Wmax & 30min simulated time-trial at 75%Wmax & 12min simulated time-trial at 85%Wmax	time (s)	↑
Gonzales et al. (2014)	10 males	30°C 79%RH	①	forehead torso	ice vest & ice head-band	cycling	EWT	20min time-trial	MPO (W)	↑*
Morrison et al. (2014)	10 males	30°C 50%RH	③	torso four limbs	CWI	cycling	ETE	exercise to exhaustion at 95%VT	duration (min)	↑**
Taylor et al. (2014)	8 females	35°C 60%RH	②	lower limbs	CWI	rowing	TT	2km time-trial	time (min)	↑
Faulkner et al. (2015)	10 males	35°C 51%RH	③	torso arms	cooling garment	cycling	TT	60min simulated time-trial at 75%Wmax	MPO (W)	↑
Faulkner et al. (2015)	10 males	35°C 51%RH	③	torso arms	cooling garment	cycling	TT	60min simulated time-trial at 75%Wmax	MPO (W)	↑*
James et al. (2015)	12 males	32°C 62%RH	③	head neak torso forearms hands thighs	CWI & ice vest & ice towels & ice packs	running	ETE	incremental exercise until an exponential increase in blood lactate or unable to complete the subsequent stage & incremental exercise to exhaustion	speed at VO2max (km·h^-1^)	↑
Randall et al. (2015)	8 males	32°C 49%RH	①	torso	ice vest	running	TT	5km time-trial	time (min)	↑
Randall et al. (2015)	8 males	32°C 49%RH	②	thighs	ice packs	running	TT	5km time-trial	time (min)	↑*
Sunderland et al. (2015)	7 males	33°C 53%RH	①	neck	neck-cooling collar	running	IS	2×45min football-specific intermittent sprint	MPO (W)	↑**
Azad et al. (2016)	16 males	32-34°C 50%RH	③	torso four limbs	CWI	running	ETE	incremental exercise to exhaustion	duration (min)	↑
Cuttell et al. (2016)	8 males	35°C 50%RH	①	torso	ice vest	cycling	ETE	exercise to exhaustion at 60%MPO	duration (min)	↑*
Cuttell et al. (2016)	8 males	35°C 50%RH	①	neck	neck-cooling collar	cycling	ETE	exercise to exhaustion at 60%MPO	duration (min)	↑
Maia-Lima et al. (2017)	8 males	35°C 68%RH	③	torso four limbs	CWI	cycling	TT	30km time-trial	time (min)	↑*
Ruddock et al. (2017)	9 males	35°C 50%RH	②	hands	CWI	cycling	ETE	exercise to exhaustion at 50%VO2peak	duration (min)	↑
Ruddock et al. (2017)	9 males	35°C 50%RH	②	hands	CWI	cycling	ETE	exercise to exhaustion at 50%VO2peak	duration (min)	↑
Schmit et al. (2017)	13 males	35°C 50%RH	①	torso	ice vest	cycling	TT	20km time-trial	time (s)	↑
Walters et al. (2017)	22 males	35°C 15%RH	①	head	head-cooling device	cycling	ETE	40min at 65%VO2peak & incremental exercise to exhaustion	PPO (W)	↑**
James et al. (2018)	8 males and 1 female	32°C 60%RH	③	head neak torso forearms hands thighs	CWI & ice vest & ice towels & ice packs	running	TT	5km time-trial	time (s)	↑*
Katica et al. (2018)	8 males	35°C 44%RH	①	head neak torso	ice vest & ice wraps	cycling	TT	16.1km time-trial	time (min)	↑*
Maroni et al. (2018)	12 males	35°C 53%RH	②	dominant hand	cooling glove	cycling	IS	2×30min intermittent sprint	MPO (W)	↑
Maroni et al. (2018)	12 males	35°C 53%RH	①	torso	cooling jacket	cycling	IS	2×30min intermittent sprint	MPO (W)	↑
Maroni et al. (2018)	12 males	35°C 53%RH	③	torso dominant hand	cooling jacket & cooling glove	cycling	IS	2×30min intermittent sprint	MPO (W)	↑
Smith et al. (2018)	13 males and 13 females	32°C 59%RH	③	head neak torso thighs	heat emergency kit	running	TT	30min self-paced exercise & 1.5km time-trial	time (min)	↑
Chaen et al. (2019)	8 males	33°C 50%RH	①	torso	ice vest	cycling	IS	2×30min intermittent sprint	MPO (W)	↑*
Chan et al. (2019)	10 males	33°C 75%RH	①	torso	hybrid cooling vest	running	ETE	incremental exercise to exhaustion	duration (min)	↑
Choo et al. (2019)	11 males	34°C 43%RH	③	torso four limbs	CWI	cycling	EWT	60 min at RPE=15	total work done (kJ)	↑*
Faulkner et al. (2019)	8 males	35°C 51%RH	③	torso arms	cooling garment	cycling	TT	60min simulated time-trial at 75%Wmax	time (s)	↑*
Maroni et al. (2020)	10 males	35°C 57%RH	②	hands	cooling glove	cycling	IS	43min intermittent sprint	total work done (kJ)	↑
Maroni et al. (2020)	10 males	35°C 57%RH	①	torso	cooling jacket	cycling	IS	43min intermittent sprint	total work done (kJ)	↑
Maroni et al. (2020)	10 males	35°C 57%RH	③	torso hands	cooling jacket & cooling glove	cycling	IS	43min intermittent sprint	total work done (kJ)	↑
Thomas et al. (2019)	10 males	34°C 36%RH	③	torso arms	ice vest & ice towels	running	IS	46min intermittent sprint	distance (km)	↑
Wohlfert and Miller (2019)	6 males and 6 females	39°C 36%RH	③	torso four limbs	CWI	running	ETE	exercise to exhaustion (walking for 3min at 3mph & running for 2min at 90%HRmax)	duration (min)	↑**
Nakamura et al. (2020)	8 males	35°C 60%RH	②	forearms hands	CWI	cycling	ETE	exercise at 55%VO2max until 38.5°C (core temperature) & exercise to exhaustion at 75%VO2max	duration (min)	↑*
Coelho et al. (2021)	15 males	35°C 50%RH	①	head	cooling cap	running	TT	5km time-trial	time (s)	↑*
Filep et al. (2021)	11 males	35°C 50%RH	②	forearms	CWI	running	TT	6×20min exercise & 1.61km time-trial	time (s)	↑*
Moss et al. (2021)	9 males	40°C 50%RH	①	neck	neck-cooling collar	cycling	EWT	15min time-trial	distance (km)	↔
Osakabe et al. (2021)	8 males	35°C 50%RH	③	face neck arms thighs	fan & wet sponge	cycling	IS	2×30min intermittent sprint	MPO (W)	↓
Xu et al. (2021)	7 males	38°C 55%RH	①	torso	ice vest	running	ETE	exercise to exhaustion at 80%VO2max	duration (s)	↑
Naito et al. (2022)	4 males and 4 females	34°C 49%RH	①	torso	ice vest	running	EWT	3 sets outdoor match-play tennis	distance (m)	↑

RH: relative humidity; ①: central cooling; ②: peripheral cooling; ③: central and peripheral cooling; CWI: cold water immersion; IS: intermittent sprint; ETE: exercise to exhaustion; EWT: exercise within the fixed time or sets; TT: time-trial; PPO: peak power output; MPO: mean power output; RPO: relative power output; ↑: increased compared with control; ↓: decreased compared with control; ⟷: no change compared with control; *: *p*≦0.05; **: *p*≦0.01.

### Quantitative analysis

The sport performance tests in included studies can be categorized into four subgroups, namely: IS, ETE, EWT and TT. On the basis of different external cooling placements (central cooling, peripheral cooling and central and peripheral cooling), quantitative analysing the effects of each cooling placement on four sport performance tests. In general, the effect of central cooling was relatively best (SMD = 0.43, 95% CI 0.27 to 0.58, *p* < 0.001), followed by central and peripheral cooling (SMD = 0.39, 95% CI 0.23 to .55, *p* < 0.001), and the effect of peripheral cooling was the relatively worst (SMD = 0.32, 95% CI 0.07 to 0.57, *p* = 0.013).

#### Effects of central cooling on athletic performance

**FIGURE 2:**
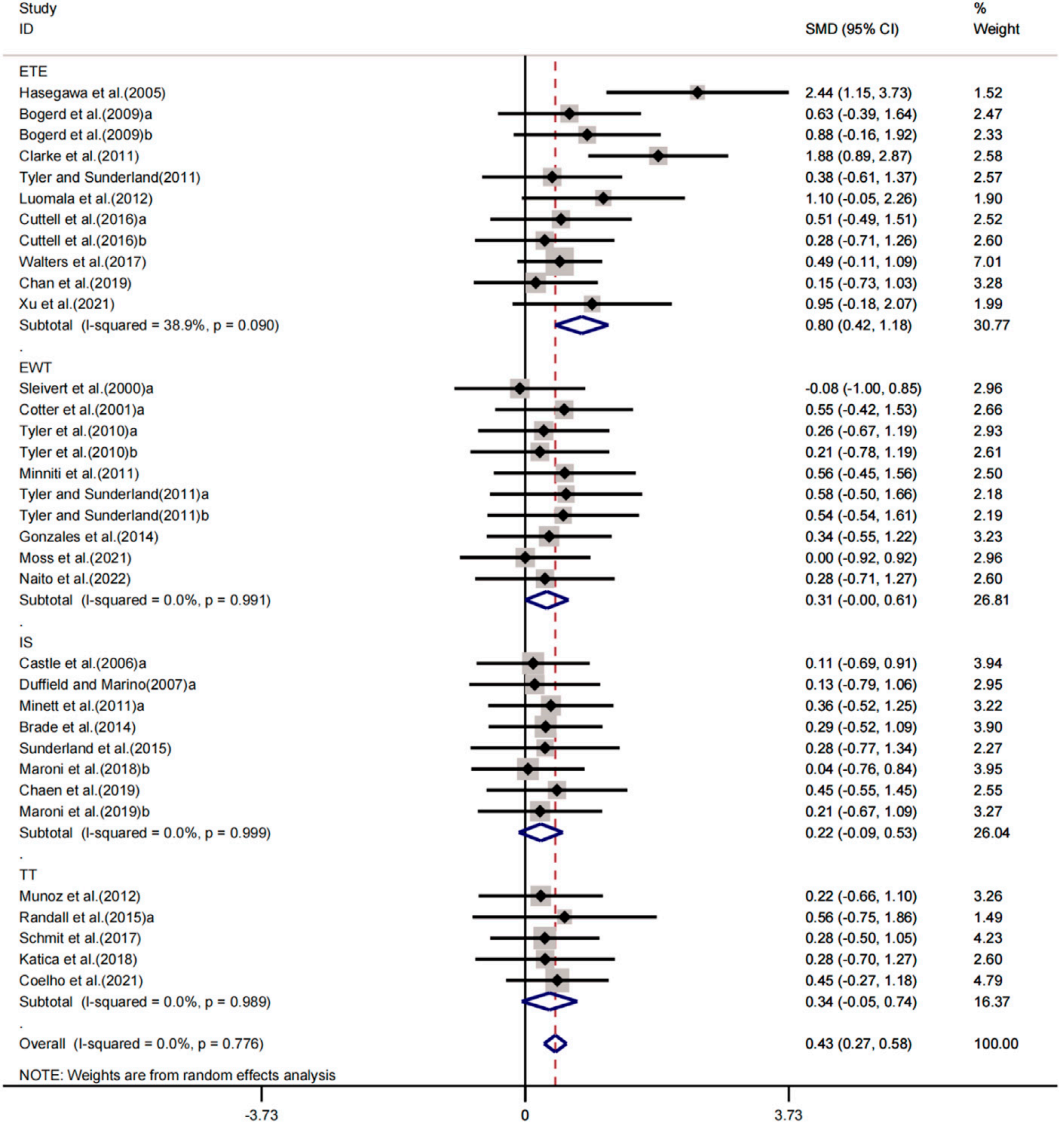
Forest plot regarding the effects of central cooling on athletic performance.

**FIGURE 3:**
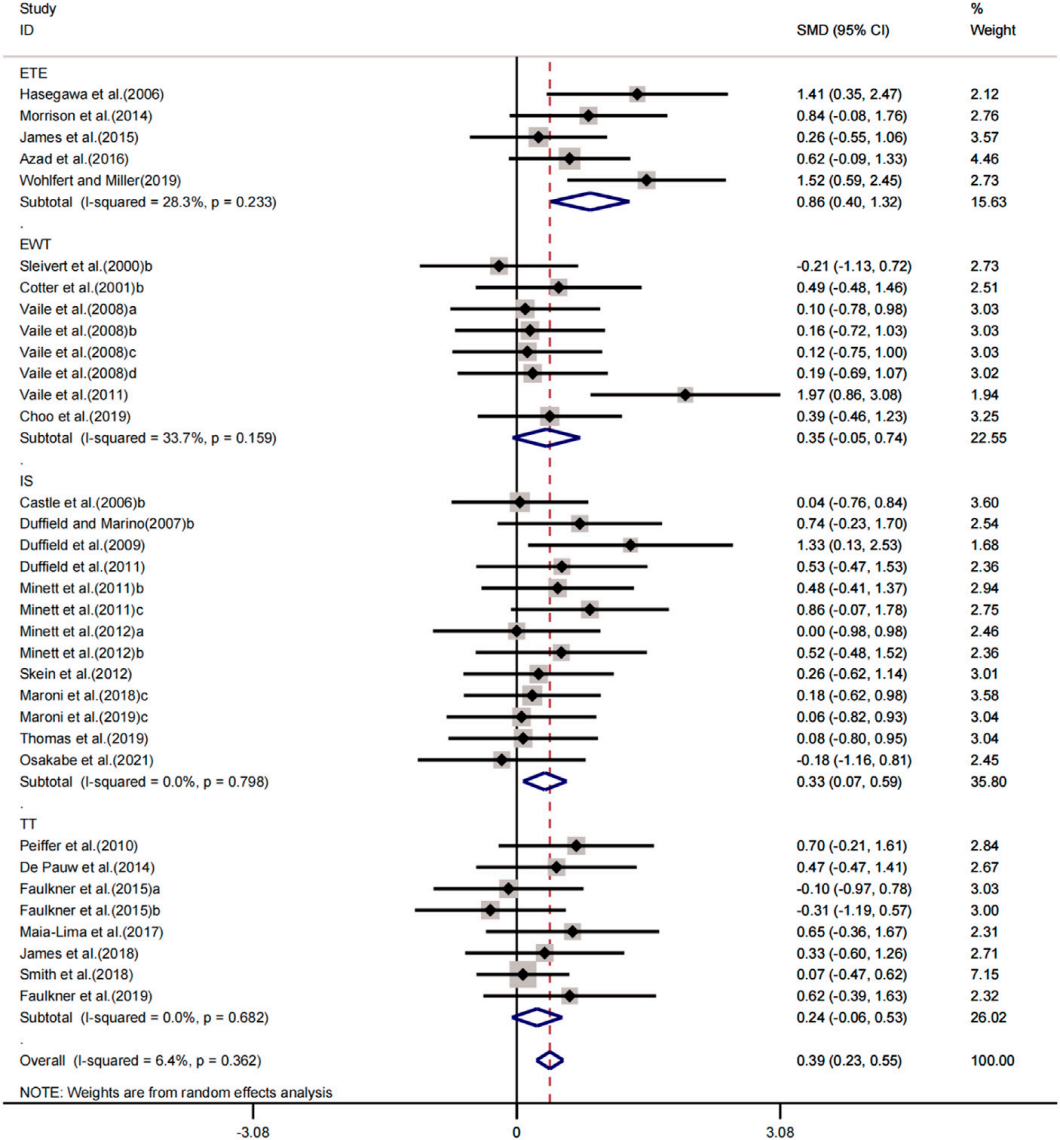
Funnel plots of publication bias for athletic performance.

In a meta-analysis including 34 experiments, we found that central cooling interventions significantly enhanced sport performance in the heat. The pooled effect size was significant with small ES (SMD = 0.43, 95% CI 0.27 to 0.58, *p* < 0.001, Figure 2) and no heterogeneity (I^2^ = 0.0%, *p* = 0.776). The funnel plot (Figures 3A) and Egger’s test (t = 2.61, *p* = 0.014) indicated that there was publication bias on these results. Processed by the Trim and Fill method, analysis results showed that seven studies were added and the pooled ES was robust (SMD = 0.527, 95% CI 0.384 to .671, *p* < 0.001) compared to before trim and fill.

Sub-group analysing for the four exercise protocols (IS, ETE, EWT and TT) showed that central cooling interventions had small, positive and statically insignificant effects on IS (SMD = 0.22, 95% CI -0.09 to 0.53, *p* = 0.165) without heterogeneity (I^2^ = 0.0%, *p* = 0.999); moderate, positive and statically significant effects on ETE (SMD = 0.80, 95% CI 0.42 to 1.18, *p* < 0.001) with low heterogeneity (I^2^ = 38.9%, *p* = 0.090); small, positive and statically insignificant effects on EWT (SMD = 0.31, 95% CI -0.00 to 0.61, *p* = 0.051) without heterogeneity (I^2^ = 0.0%, *p* = 0.991); small, positive and statically insignificant effects on TT (SMD = 0.34, 95% CI -0.05 to 0.74, *p* = 0.086) without heterogeneity (I^2^ = 0.0%, *p* = 0.989).

#### Effects of peripheral cooling on athletic performance

**FIGURE 4:**
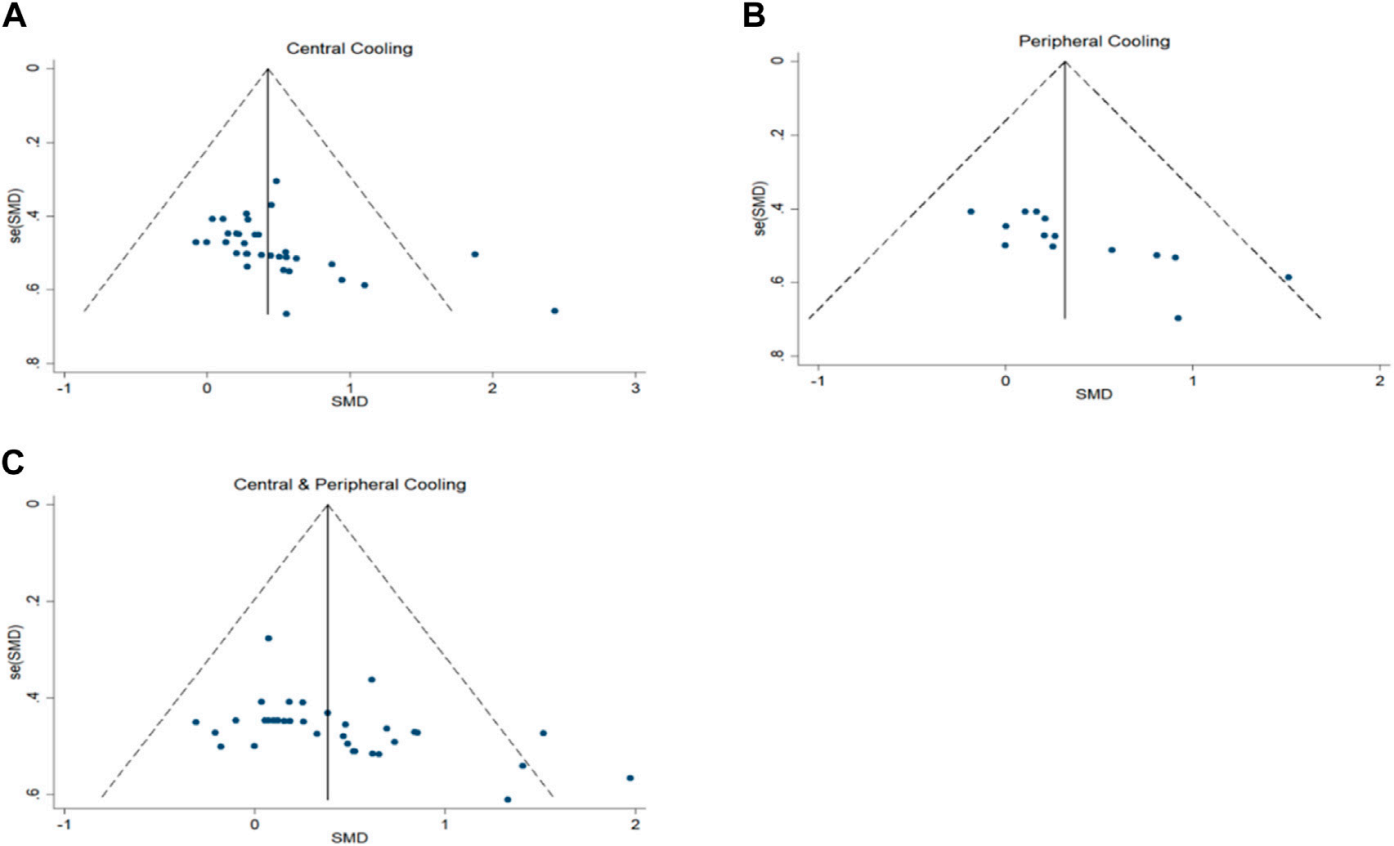
Forest plot regarding the effects of peripheral cooling on athletic performance.

In a meta-analysis including 14 experiments, we found that peripheral cooling interventions significantly enhanced sport performance in the heat. The pooled effect size was significant with small ES (SMD = 0.32, 95% CI 0.07 to 0.57, *p* = 0.013, Figure 4) and no heterogeneity (I^2^ = 0.0%, *p* = 0.680). The funnel plot (Figures 3B) and Egger’s test (t = 4.37, *p* = 0.001) indicated that there was publication bias on these results. Processed by the Trim and Fill method, analysis results showed that one studies were added and the pooled ES was robust (SMD = 1.296, 95% CI 1.014 to 1.656, *p* = 0.038) compared to before trim and fill.

Sub-group analysing for the four exercise protocols (IS, ETE, EWT and TT) showed that peripheral cooling interventions had trivial, positive and statically insignificant effects on IS (SMD = 0.08, 95% CI -0.35 to 0.51, *p* = 0.714) without heterogeneity (I^2^ = 0.0%, *p* = 0.991); small, positive and statically insignificant effects on ETE (SMD = 0.37, 95% CI -0.28 to 1.02, *p* = 0.265) with low heterogeneity (I^2^ = 47.3%, *p* = 0.127); large, positive and statically significant effects on EWT (SMD = 0.86, 95% CI 0.13 to 1.59, *p* = 0.022) without heterogeneity (I^2^ = 0.0%, *p* = 0.894); small, positive and statically insignificant effects on TT (SMD = 0.41, 95% CI -0.09 to 0.91, *p* = 0.109) without heterogeneity (I^2^ = 0.0%, *p* = 0.815).

#### Effects of central and peripheral cooling on athletic performance

**FIGURE 5:**
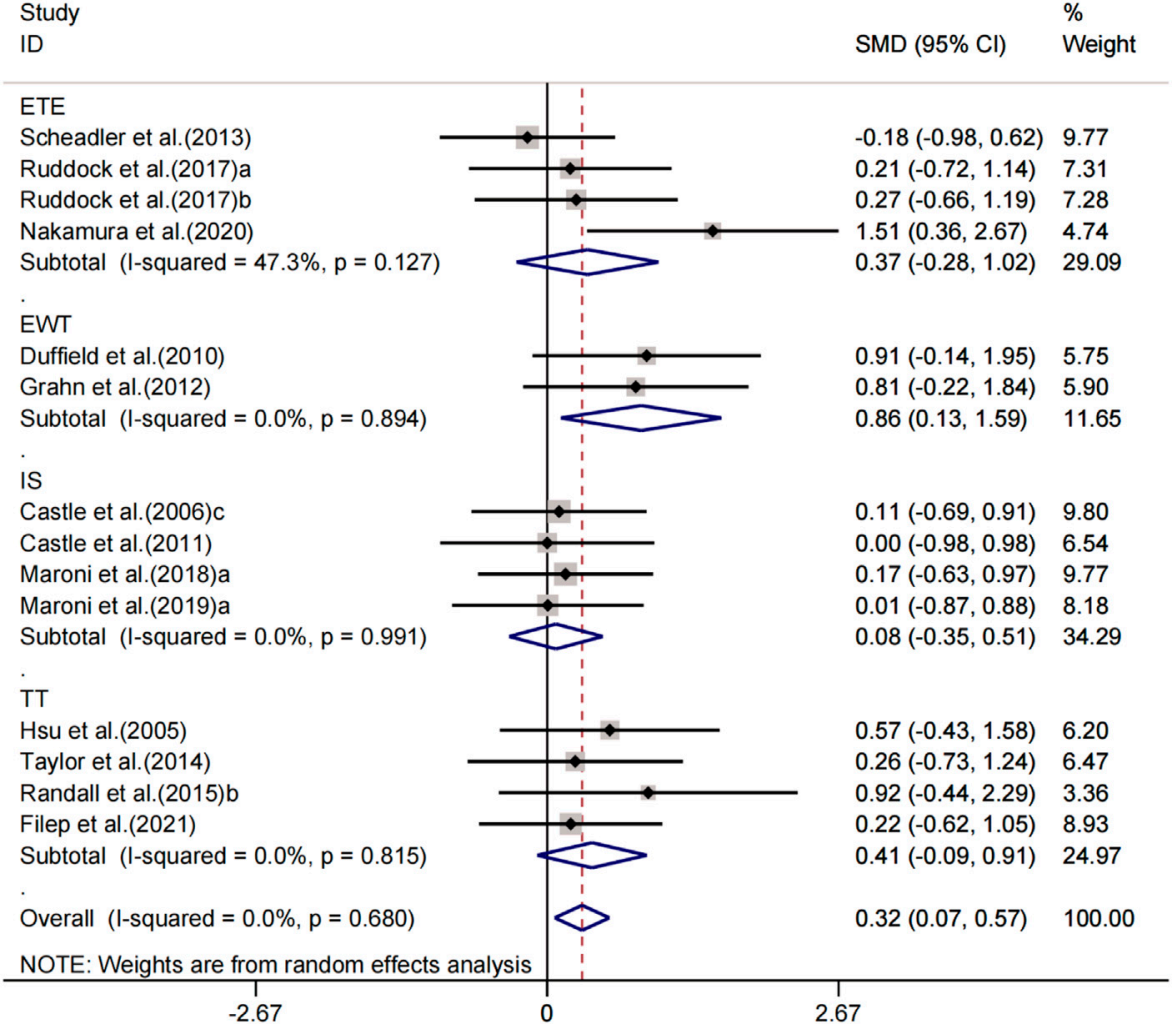
Forest plot regarding the effects of central and peripheral cooling on athletic performance

In a meta-analysis including 34 experiments, we found that central and peripheral cooling interventions significantly enhanced sport performance in the heat. The pooled effect size was significant with small ES (SMD = .39, 95% CI .23 to .55, *p* < 0.001, Figure 5) and low heterogeneity (I^2^ = 6.4%, *p* = 0.362). The funnel plot (Figures 3C) and Egger’s test (t = 2.87, *p* = 0.007) indicated that there was publication bias on these results. Processed by the Trim and Fill method, analysis results showed that no study was added and the pooled ES was robust (SMD = 1.468, 95% CI 1.260 to 1.709, *p* < 0.001) compared to before trim and fill.

Sub-group analysing for the four exercise protocols (IS, ETE, EWT and TT) showed that central and peripheral cooling interventions had small, positive and statically significant effects on IS (SMD = 0.33, 95% CI 0.07 to 0.59, *p* = 0.012) without heterogeneity (I^2^ = 0.0%, *p* = 0.798); large, positive and statically significant effects on ETE (SMD = 0.86, 95% CI 0.40 to 1.32, *p* < 0.001) with low heterogeneity (I^2^ = 28.3%, *p* = 0.233); small, positive and statically significant effects on EWT (SMD = 0.35, 95% CI -0.05 to 0.74, *p* = 0.086) with low heterogeneity (I^2^ = 33.7%, *p* = 0.159); small, positive and statically insignificant effects on TT (SMD = 0.24, 95% CI -0.06 to 0.53, *p* = 0.115) without heterogeneity (I^2^ = 0.0%, *p* = 0.682).

## Discussion

### Effects of central cooling on athletic performance

Central cooling showed the optimal effects on the athletic performance, compared to the other two cooling strategies, on the basis of the research results of this study. Central cooling can be further divided into torso cooling and neck and head cooling by different functional characteristics. On the one hand, torso cooling, such as cooling vest, has a beneficial effect on skin temperatures, resulting in improved exercise performance (Ross et al., 2013; Chaen et al., 2019). During strenuous exercise in the heat, there exists a fierce competition for blood flow between the skin and muscles to the extent that muscle blood flow can be impaired (González-Alonso et al., 1999), which may induce negative effects on supply of energy substrates and removal of metabolic waste, and ultimately lead to premature fatigue. The reduction in torso skin temperature can decrease the peripheral skin blood flow and increase blood supply to the skeletal muscle (James et al., 2015) *via* superficial blood vessel constriction, and expand the heat gradient from the core to the skin to help promoting heat loss and reducing cardiovascular strain (Cheuvront et al., 2010). Mitigation of competition pressure between the skin and muscles, possibly allowing better maintenance of venous return, cardiac output and mean arterial pressure, and greater blood flow perfusion of the active muscle, thereby boosting oxygen delivery or metabolite removal, and increasing the contribution of aerobic metabolism to ATP re-phosphorylation (Marsh and Sleivert, 1999; Cotter et al., 2001). On the other hand, as neck and head are adjacent to the temperature-regulating centers and high sensory thermoreceptor, which dominate whole-body temperature perception (Cotter and Taylor, 2005; Kim et al., 2019), neck and head cooling could alter the sensory feedback and attenuate the inhibitory effect on neural drive (Watkins et al., 2018), thereby changing thermal sensation, mitigating central fatigue and improving exercise performance in the heat (Chaen et al., 2019; Cao et al., 2022). However, central cooling seems not to be able to significantly alter the physiological or peripheral neuroendocrinological responses to the exercise in the heat (Tyler et al., 2010). Based on above evidences, we concluded that central cooling could improve exercise performance with improvements of skin temperature and thermal sensation, which allowed the athletes to tolerate higher core temperature and HR induced by high performance in the heat (Tyler and Sunderland, 2011).

In terms of the effects on different sports types, the ranking as follows: ETE, TT, EWT and IS. Duffield and Marino (2007) reported that it is possible that the effects of pre-cooling (ice-vest and ice-bath) on IS performance are minor and only manifest when the thermal and exercise stress is sufficient to induce heat strain. Therefore, it seems that the extent of heat strain during exercise in the heat could influence the effects of central cooling. Because that ETE requires athletes exercise at a fixed load until exhaustion without interval recovery, stimulating the physiological limits of athletes, which may elicit greater thermoregulatory and physiological strain, ETE is more likely to gain more benefits from central cooling through improvements from skin temperature and thermal sensation. By contrast, as the intensity and duration of other three exercise type (TT, EWT and IS) are optional, it is difficult to guarantee that athletes suffer sufficient heat strain and even approach to their physiological limits. Moreover, different from the continuous exercise of ETE, IS allows athletes to have interval-recovery, which may further weaken the dependence on mitigating heat strain through central cooling. Hence, it is suggested that the implementation of central cooling depends on the situation, and this method can bring more benefits to the exercise with more heat strain, such as ETE.

### Effects of peripheral cooling on athletic performance

Peripheral cooling showed the least effects on the athletic performance, compared to the other two cooling strategies, on the basis of the research results of this study. The critical limiting factor for exercise performance in the heat is an elevated core body temperature (Maughan, 2010), and accumulation of body heat mostly comes from metabolic heat production from working muscle contraction (Taylor et al., 2014). Furthermore, research also showed that significantly shorter endurance times were found for the heated muscle, because that the accumulation of hydrogen ions in muscle caused partial inhibition of the rate controlling enzyme phosphofructokinase (Edwards et al., 1972). Castle et al. (2006) and Duffield et al. (2010) found that the effect of reducing muscle temperature in peripheral cooling was better than central cooling (Castle et al., 2006; Duffield et al., 2010). In addition, after peripheral cooling (hands and forearms cooling), the cooler blood returned to the central circulation (Livingstone et al., 1989), which helped to decrease thermal perception, attenuate an increase in body temperature, limit heat storage and lower cardiorespiratory and skin blood flow demands (Ruddock et al., 2017). Nevertheless, the results of both Castle et al. (2006) and Maroni et al. (2018) showed that both central cooling and peripheral cooling could effectively diminish the core temperature compared with passive non-cooling intervention, and central cooling was even slightly better, but no significant difference between the cooling effects on two intervention methods. Ruddock et al. (2017) also pointed out that the majority of per-cooling methods had limited influence on body temperature and performance improvements might be attributed to a lower perception of heat strain, a mismatch between thermal perception and thermal strain might impair performance and as exercise continued accelerated hyperthermia-mediated fatigue. In addition, as the sites of peripheral cooling is far from the temperature-regulating centers and high sensory thermoreceptor, the effects of mitigating thermal perception may be weakened. Hence, we deduced that the major difference between peripheral cooling and central cooling might lie in the degree of improvements in thermal perception, which caused the difference in the effects on the athletic performance.

In terms of the effects on different sports types, the ranking as follows: EWT, TT, ETE and IS. Sleivert et al. (2001) found that thighs cooling impaired peak and short-term (45s) high-intensity power outputs, as a decreased muscle temperature made direct effects on contractile function or anaerobic metabolism. Anaerobic glycolysis was less active in cooled muscle, and glycogenolysis was increased in warmed muscle (Febbraio et al., 1996). Moreover, cooling leads to changes in the activation of motor units in the working muscle, some slower motor units are recruited to sustain a given level of power output in order to compensate for the reduced power of the cooled contractile elements, which impairs maximal power output (Sleivert et al., 2001). Whereas, when a short warm-up exercise and thighs cooling were combined, detrimental effects of a cold muscle on performance would be removed by increasing muscle temperature (Sleivert et al., 2001). Thus, it was suggested that direct cooling on the working muscle might influence the subsequent high-intensity exercise performance and sufficient re-warming was required (Chaen et al., 2019). Therefore, we deduced that enhancement effects of peripheral cooling required a threshold for muscle temperature, and long term sustained endurance exercises, such as ETE, with sufficient heat strains were more likely to be strengthen when combined with peripheral cooling. In the contrast, as IS allowed athletes to have a rest to relieve hyperthermia and had certain requirements for power output, the application of peripheral cooling in IS should be treated with caution, which was also consistent with the results of this study. Because that the intensity and duration of TT and EWT are optional, which requires different characteristics of energy metabolism, it is uncertain that the exact effects of peripheral cooling on them, so future research is needed to thoroughly investigate this issue. It is suggested that developing specific peripheral cooling and warm-up protocols to prompt performance, according to the intensity and duration of exercise.

#### Effects of central and peripheral cooling on athletic performance

The results of this study showed that central and peripheral cooling was better than peripheral cooling but not as good as central cooling. Minett et al. (2011) found that 20 min of whole-body pre-cooling was more effective than head refreshing alone in intermittent sprint bouts. However, Maroni et al. (2019) denoted that there were no differences in performance when comparing a hand-cooling glove technique with a cooling jacket or a combination of both during high intensity prolonged repeated-sprint efforts in cyclists. As a result, whether a mixed external cooling method is better than an isolated cooling technique is still uncertain. Nevertheless, we inferred that central and peripheral cooling seemed to retain central cooling’s advantages of reducing physiological stress and relieving thermal perception, because that both of them showed the better promotion effects on ETE than other exercise types. Based on the previous analysis, the effects of peripheral cooling seems to be variable and may be influenced by many factors, such as initial muscle temperature, cooling temperature and so on, thus some inappropriate operations can offset the positive effects from central cooling, which leads to the effects and mechanism of the central and peripheral cooling method is uncertain.

## Limitations

The main limitation of our study is the lack of the changes in physiological parameters after different cooling interventions. This would help us to further understand how the improvements from nervous system, cardiovascular system, skeletal muscle system and other human systems or tissues transfer into athletic performance in the heat. The second limitation lies on the division of cooling placements is not detailed enough, central cooling could be further divided into face, neck, head or torso cooling, peripheral cooling also could be further divided into upper limbs or lower limbs cooling. However, because there are so many types of cooling placements, it is extremely difficult to explore the effectiveness of all types of external cooling, this article only divides external cooling into three categories and makes a rough comparison between them. Another limitation may be that the different intensity and duration of exercise need specific peripheral cooling and warm-up protocol, hence it is difficult to precisely investigate the cooling effects on these four exercise type, and even underlying mechanism behind them. In the future, the above aspects need to be further explored to provide meaningful suggestions for coaches and athletes.

## Conclusion

On the basis of the results of this meta-analysis, central cooling appears to be an more effective intervention to enhance performance in hot conditions, compared to other external cooling strategies. Central cooling can improve exercise performance with improvements of skin temperature and thermal sensation, which allows the athletes to tolerate higher physiological strains induced by high performance in the heat, and shows the best enhancement effect on ETE. The effects of peripheral cooling on IS are trivial, and the enhancement effects of peripheral cooling require sufficient re-warming, hence it is suggested that developing specific peripheral cooling and warm-up protocols to prompt performance, according to the intensity and duration of exercise. The effect of central and peripheral cooling is better than peripheral cooling but not as good as central cooling. Although, central and peripheral cooling seems to retain central cooling’s advantages of reducing physiological stress and relieving thermal perception, and both of them show the better promotion effects on ETE, as many factors may influence the effects of peripheral cooling, thus some inappropriate operations can offset the positive effects from central cooling. Hence, in the future, more researchs are needed to further explore whether a mixed external cooling method is better than an isolated cooling technique.

## Data Availability

The original contributions presented in the study are included in the article/Supplementary Material, further inquiries can be directed to the corresponding author.
