# Supplementary material for: Effects of different external cooling placements prior to and during exercise on athletic performance in the heat: A systematic review and meta-analysis
Source: Front Physiol. 2023 Jan 10;13:1091228. doi: 10.3389/fphys.2022.1091228 (PMC9871495; doi:10.3389/fphys.2022.1091228)
Supplement: Supplementary file 2 [file DataSheet1.docx]

# Supplementary Material 1:

**Search Strategy**

| Process | Keywords |
| --- | --- |
| # 1 | "Cooling" Or "External Cooling" Or "Cooling Jackets" Or "Cooling Vests" Or "Ice Vest" Or "Head Cooling" Or "Face Cooling" Or "Neck Cooling" Or "Arm Cooling" Or "Hand Cooling" Or "Leg Cooling" Or "Trunk Cooling" Or "Body Cooling" Or "Cold" Or "Cold Jacket" Or "Cold Vest" Or "Cold Water Immersion" |
| # 2 | "Athletic Performance" Or "Exercise Performance" Or "Sports Performance" Or "Exercise Capacity" |
| # 3 | "Hot" Or "Heat" Or "Hot Temperature" Or "Extreme Heat" |
| # 4 | # 1 AND # 2 AND # 3 |
